# Spatial Cognitive EEG Feature Extraction and Classification Based on MSSECNN and PCMI

**DOI:** 10.3390/bioengineering12010025

**Published:** 2024-12-31

**Authors:** Xianglong Wan, Yue Sun, Yiduo Yao, Wan Zuha Wan Hasan, Dong Wen

**Affiliations:** 1 School of Intelligence Science and Technology, University of Science and Technology Beijing, Beijing 100083, China; 2 Key Laboratory of Perception and Control of Intelligent Bionic Unmanned Systems, Ministry of Education, Institute of Artificial Intelligence, University of Science and Technology Beijing, Beijing 100083, China; 3 Department of Electrical and Electronic Engineering, Faculty of Engineering, Universiti Putra Malaysia, Serdang 43400, Malaysia

**Keywords:** spatial cognition, EEG, multi-scale convolutional neural network, squeezed excitation network, permutation conditional mutual information

## Abstract

With the aging population rising, the decline in spatial cognitive ability has become a critical issue affecting the quality of life among the elderly. Electroencephalogram (EEG) signal analysis presents substantial potential in spatial cognitive assessments. However, conventional methods struggle to effectively classify spatial cognitive states, particularly in tasks requiring multi-class discrimination of pre- and post-training cognitive states. This study proposes a novel approach for EEG signal classification, utilizing Permutation Conditional Mutual Information (PCMI) for feature extraction and a Multi-Scale Squeezed Excitation Convolutional Neural Network (MSSECNN) model for classification. Specifically, the MSSECNN classifies spatial cognitive states into two classes—before and after cognitive training—based on EEG features. First, the PCMI extracts nonlinear spatial features, generating spatial feature matrices across different channels. SENet then adaptively weights these features, highlighting key channels. Finally, the MSCNN model captures local and global features using convolution kernels of varying sizes, enhancing classification accuracy and robustness. This study systematically validates the model using cognitive training data from a brain-controlled car and manually operated UAV tasks, with cognitive state assessments performed through spatial cognition games combined with EEG signals. The experimental findings demonstrate that the proposed model significantly outperforms traditional methods, offering superior classification accuracy, robustness, and feature extraction capabilities. The MSSECNN model’s advantages in spatial cognitive state classification provide valuable technical support for early identification and intervention in cognitive decline.

## 1. Introduction

With the intensifying global trend of population aging, the decline in spatial cognitive abilities has emerged as a critical issue affecting the quality of life among the elderly [1]. Studies have shown that the deterioration of spatial cognitive abilities is closely related to early symptoms of neurodegenerative diseases such as Alzheimer’s [2,3], Parkinson’s [4] and Dementia with Lewy Bodies [5]. In neuroscience and cognitive science, EEG signal analysis has become an essential tool for assessing individual cognitive function [6,7]. EEG signals can reflect the brain’s responses to various cognitive tasks by analyzing activities across different frequency bands. This study focuses on classifying cognitive states before and after spatial cognitive training, a crucial step for assessing the effectiveness of such training interventions and monitoring changes in cognitive function [8,9,10]. Therefore, evaluating spatial cognition through EEG signals holds significant promise for the early detection and intervention of cognitive decline [11,12].

Traditional methods for EEG signal analysis include spectral analysis [13,14,15,16], Blind Source Separation (BSS) [17,18], brain region synchronization analysis [19,20,21,22,23], Event-Related Potentials (ERPs) [24,25,26], and brain functional network analysis [27,28]. These approaches mainly investigate EEG signal features in the time and frequency domains. However, they often fail to capture the spatial features of EEG signals in sufficient detail, especially under complex cognitive tasks, making it challenging to extract the multi-scale spatial features of EEG comprehensively [29]. Furthermore, although convolutional neural networks (CNNs) have shown success in EEG signal classification, the fixed size of convolutional kernels limits the effective extraction of multi-scale features, which can result in feature information loss [30,31]. Addressing how to effectively integrate multi-scale spatial features of EEG signals to enhance classification accuracy and model robustness remains a critical challenge in spatial cognitive assessment, particularly in distinguishing pre-training and post-training cognitive states during spatial cognition tasks.

In EEG signal analysis for spatial cognitive assessment, researchers have traditionally employed the Common Spatial Pattern (CSP) algorithm to extract spatial features, achieving notable success, particularly in binary classification tasks [32]. CSP captures variations in EEG activity by extracting spatial features of signals in different states. However, due to CSP’s reliance on linear assumptions, it often needs help to handle the nonlinear features of EEG signals effectively. To address this limitation, researchers have introduced methods such as Mutual Information (MI) and Conditional Mutual Information (CMI), which have nonlinear feature extraction capabilities [33]. However, MI and CMI are susceptible to noise [34], limiting their practical applications. The improved Permutation Conditional Mutual Information (PCMI) has shown excellent performance in quantifying coupling strength between time series, with enhanced robustness in noisy environments [35,36]. Studies indicate that PCMI is advantageous in extracting EEG features before and after spatial cognitive training, offering theoretical support for using PCMI in spatial cognitive EEG analysis [37,38].

Although CNNs are traditionally applied to image data, their ability to extract hierarchical spatial features makes them well-suited for EEG analysis when the signals are transformed into structured spatial feature matrices. By representing EEG signals as channel matrices derived from PCMI, CNNs can leverage their convolutional layers to capture both local and global spatial dependencies across brain regions. This approach complements the temporal resolution of EEG signals and enhances classification performance in spatial cognition tasks [39,40]. The MSCNN overcomes the limitations of traditional CNNs in handling scale variation by extracting local and global features using convolutional kernels of different sizes [41,42,43]. However, existing multi-scale approaches still face challenges related to feature weight imbalance in small-sample conditions [44], which affects classification performance. CNNs have limited ability to weight feature channels, which prevents the model from effectively leveraging the varying contributions of different channels, thereby reducing classification accuracy and robustness.

To address these challenges, researchers proposed SENet [45], which enhances the response of essential feature channels through adaptive weighting, thereby improving classification performance. Although integrating SENet and the MSCNN has been preliminarily explored, comprehensively utilizing multi-scale features and significant channel weighting in EEG signals remains an open problem.

To tackle this problem, this study proposes the MSSECNN model, which focuses on classifying cognitive states into two categories: pre-training and post-training. The model employs PCMI for effective feature extraction and combines SENet with the MSCNN to enhance classification accuracy, particularly for EEG signals collected during spatial cognition tasks. Through the combined effects of PCMI and SENet, the model accurately extracts key features from multi-frequency band EEG signals. At the same time, the MSCNN enables comprehensive multi-scale information capture, significantly enhancing classification accuracy and robustness. Specifically, the central processing steps of the MSSECNN model include:

(1) Extracting nonlinear spatial features from EEG signals using the PCMI method to generate spatial feature matrices across different frequency bands;

(2) Applying SENet for adaptive weighting of the extracted PCMI features, highlighting key feature channels;

(3) Feeding the weighted features into the MSCNN model, which utilizes convolutional kernels of varying sizes to further capture local and global features, ensuring the acquisition of multi-level features.

The model was validated on EEG data from a brain-controlled car and hand-controlled UAV tasks, and its effectiveness is compared with traditional CNN and MSCNN methods.

## 2. Dataset and Signal Processing Methods

### 2.1. Participant Information and Data Acquisition Process

With support from the Ethics Committee of the First People’s Hospital of Qinhuangdao, Hebei Province, 25 healthy participants (19 males and 6 females) were recruited from nearby communities, all participating voluntarily. The average age of participants was 23.76 ± 1.83 years. Based on the spatial cognitive training task requirements, participants were randomly assigned to a brain-controlled car or hand-controlled UAV group. The brain-controlled car group included 13 participants (9 males and 4 females) with an average age of 24 ± 2.08 years, while the hand-controlled UAV group included 12 participants (10 males and 2 females) with an average age of 23.5 ± 1.57 years. EEG signals were collected from these participants during spatial cognitive assessment tasks conducted before and after cognitive training. All participants signed informed consent forms before the study began, had normal or corrected-to-normal vision, no history of major psychiatric or neurological disorders, and were first-time participants in such experimental training. This study was approved by the Ethics Committee of the First People’s Hospital of Qinhuangdao (Approval No.: 2018B006).

The data collection process for this study involved three stages: Initially, baseline EEG data were recorded using a cognitive assessment system before training; secondly, participants underwent a 28-day training program involving brain-controlled car [46] and hand-controlled UAV [47]; finally, post-training EEG data were recorded using the cognitive assessment system. Statistical analyses indicated significant differences in specific EEG signal indicators before and after training (*p* < 0.05), suggesting that spatial cognitive training substantially impacted EEG activity.

**Figure 1:**
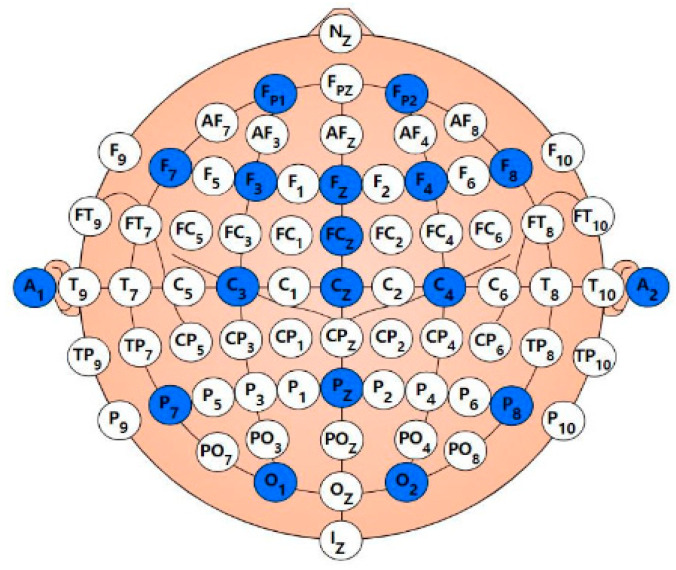
Electrode position.

To ensure the accuracy and consistency of EEG signals during data collection, strict control was maintained over equipment parameters and the data acquisition process. The EEG sampling rate was 1000 Hz, with electrode impedance at or below 10 kΩ. A 16-channel OpenBCI amplifier (manufactured by OpenBCI, New York, NY, USA) was employed for data acquisition, with data transmitted to a computer via Wi-Fi. Electrode placement was based on the study by Kenny et al. [48], following the standard 10–20 electrode placement system, which is widely used for EEG recordings. Specific electrode distributions, conforming to the 10–20 system, are shown in Figure 1, where the blue channels represent the recorded channels used in this study.

### 2.2. EEG Signal Preprocessing

During cognitive tasks, hand movements turns and other motions of participants can introduce environmental noise, while eye blinks, electromyographic (EMG), and electrocardiographic (ECG) interference can further affect EEG signal quality. The following preprocessing steps were applied to the raw EEG signals:

(1) Notch Filtering: A Type I Chebyshev bandpass filter was used to filter the signal within the 1–100 Hz frequency range, and a 50 Hz notch filter was applied to remove power line interference.

(2) Independent Component Analysis (ICA): Fast ICA removed artifacts such as ocular, muscular, and cardiac interference.

(3) Downsampling: The data sampling rate was reduced from 1000 Hz to 125 Hz.

(4) Frequency Band Segmentation: A bandpass filter was used to segment the EEG signals into seven frequency bands: Delta (1–4 Hz), Theta (4–8 Hz), Alpha1 (8–10.5 Hz), Alpha2 (10.5–13 Hz), Beta1 (13–20 Hz), Beta2 (20–30 Hz), and Gamma (30–40 Hz).

(5) Segment Extraction: During cognitive tasks, EEG signal segments were extracted using a sliding window method with a window length of 4 s, a step size of 2 s, and a 50% overlap to ensure full task coverage.

(6) Frequency Band Combination: Distinct EEG frequency bands are associated with specific cognitive processes, underscoring their relevance in task-specific brain activity analysis. Therefore, this study designed various frequency band combinations to evaluate the applicability of the MSSECNN model in spatial cognitive feature extraction. Specifically, Theta, Alpha, and Beta bands are typically associated with cognitive functions such as attention and memory, while the Gamma band reflects higher-order cognitive activities. Multi-frequency band combinations comprehensively evaluate the MSSECNN model’s feature extraction performance and classification capabilities across frequency bands, ensuring model robustness and effective multi-frequency band feature extraction.

## 3. Dataset and Signal Preprocessing Methods

### 3.1. Permutation Conditional Mutual Information

PCMI is a method for quantifying the directional information flow between time series, particularly suitable for analyzing strongly and weakly coupled time series data in complex systems [49]. In EEG signal analysis, PCMI is used to estimate the direction and intensity of information transfer between different brain regions, thereby revealing features of brain functional connectivity [50]. Studies have shown that PCMI is highly robust under low signal-to-noise ratio conditions and effectively captures nonlinear features in EEG signals [51]. Additionally, PCMI has been applied in analyzing EEG data from patients with mild cognitive impairment (MCI), aiding in MCI diagnosis by extracting coupling features [36,52,53]. Therefore, this study adopts a PCMI-based feature extraction approach, quantifying inter-regional coupling features to enable practical analysis of multi-channel EEG signals.

### 3.2. Squeezed Excitation Network

**Figure 2:**
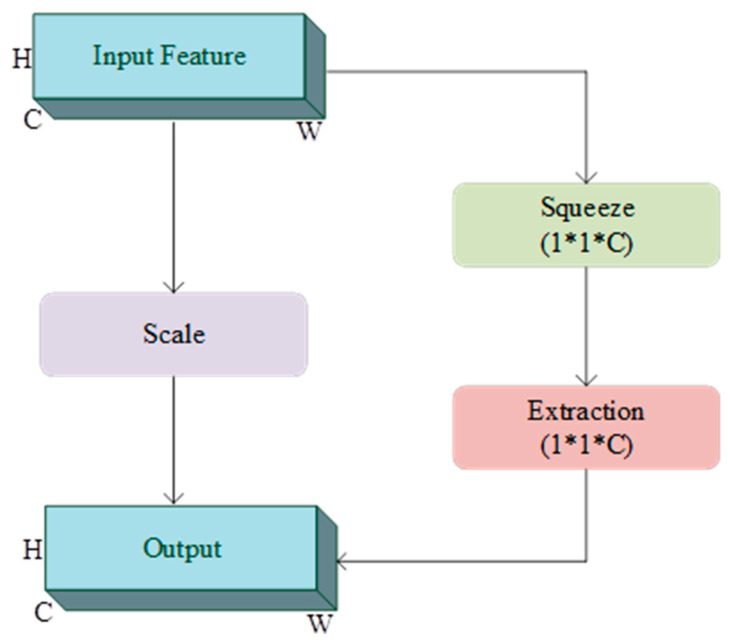
Squeezed excitation network.

This study employs SENet to enhance important feature information, improving classification accuracy and robustness. As proposed by the Momenta team, SENet can be embedded into various classification network structures, adjusting the response values of each frequency band adaptively to boost the model’s classification performance [54]. SENet models dependencies among the frequency bands in the input data, following a three-stage process: squeeze, excitation, and reweight, with its specific structure shown in Figure 2.

In SENet, the input data are represented as $X\in R^{H\times W\times C}$, where H denotes the data height, W the width, and C the number of channels. SENet performs adaptive weighting on each channel’s features through the following three steps:

Squeeze Operation: This step uses global average pooling to aggregate information from each channel into a global descriptor, reducing the spatial dimensionality of the data. Specifically, the 2D feature matrix $X\in R^{H\times W\times C}$ is compressed into a 1×1×C representation denoted as $Z_{C}$. The descriptor for each channel is obtained through global average pooling, calculated as shown in Equation (1):

(1) $$Z_{c}=F_{Squeeze}\left(x_{c}\right)=\frac{1}{H\times W}\sum_{i=1}^{W}\sum_{j=1}^{H}x_{c}(i,j)$$

Excitation Operation: This step further learns the nonlinear relationships among channels by applying two fully connected layers and assigning adaptive weights to each channel. First, the dimensionality is reduced from C to $\frac{C}{r}$ (where r is a scaling parameter) using a fully connected layer with ReLU activation. Then, a second fully connected layer restores the dimensionality to C and normalizes the output values to the [0, 1] range with a Sigmoid activation function, producing channel weights $S_{c}$. The excitation operation is calculated as shown in Equation (2):

(2) $$S_{c}=F_{excitation\;}\left(Z_{c},W\right)=\sigma \left(g\left(Z_{c},W\right)\right)=\sigma \left(W_{2}ReLU\left(W_{1}Z_{c}\right)\right)$$

$W_{1}$ and $W_{2}$ represent the weight matrices of the two fully connected layers, and σ denotes the Sigmoid activation function.

Reweighting Operation: In this stage, the learned channel weights $S_{c}$ are applied to each channel in the original input data X, enhancing the response of essential features while suppressing less relevant features. The reweighting calculation is shown in Equation (3):

(3) $${\tilde{X}}_{i,j,c}=S_{c}\cdot X_{i,j,c}$$

${\tilde{X}}_{i,j,\;c}$ represent the output data after reweighing the channel importance.

### 3.3. Multi-Scale Convolutional Neural Network

**Figure 3:**
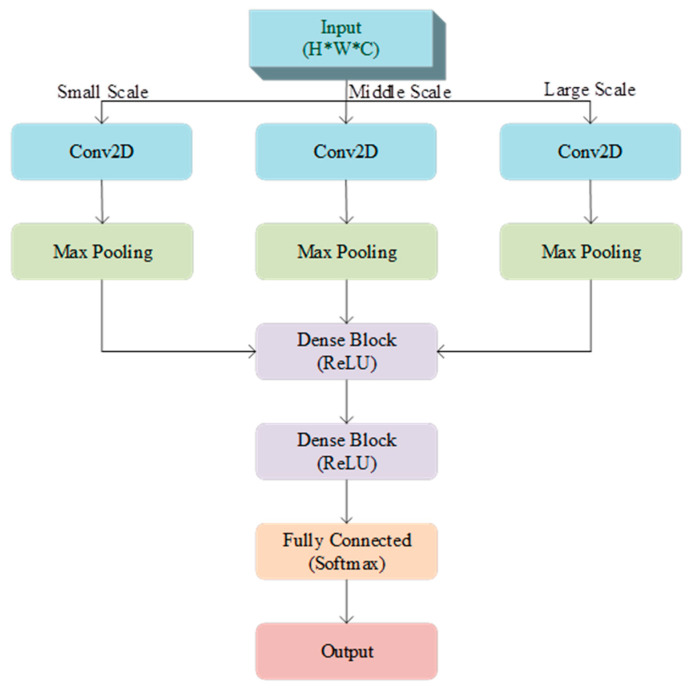
Multi-scale convolutional neural network.

The Google and Magic Leap teams initially proposed the MSCNN based on the convolutional concept of the Network in Network (NiN) architecture. It was further developed through the Inception module to create a CNN structure capable of extracting multi-scale features [45]. The MSCNN enhances the network’s ability to capture features across various scales by stacking multiple Inception modules, as shown in Figure 3.

The input to the MSCNN is a coupled feature matrix of size H×W×C. First, the input feature matrix is convolved with kernels of three different sizes to extract features at multiple scales, with a ReLU activation function applied after each convolution layer for nonlinear mapping. Subsequently, the convolution results in undergoing max pooling to reduce spatial dimensionality, lower the risk of overfitting, and retain critical feature information. The pooled results from the three convolution branches are concatenated along the channel dimension to form a multi-scale feature map, which is then inputted to the next layer. During the feature reorganization and classification stage, the concatenated feature map is fed into a densely connected layer, where ReLU activation is employed to further capture inter-feature relationships. Finally, the fully connected output layer uses a Softmax activation function for binary classification, alleviating the vanishing gradient problem and improving classification accuracy.

### 3.4. Multi-Scale Squeezed Excitation Convolutional Network

**Figure 4:**
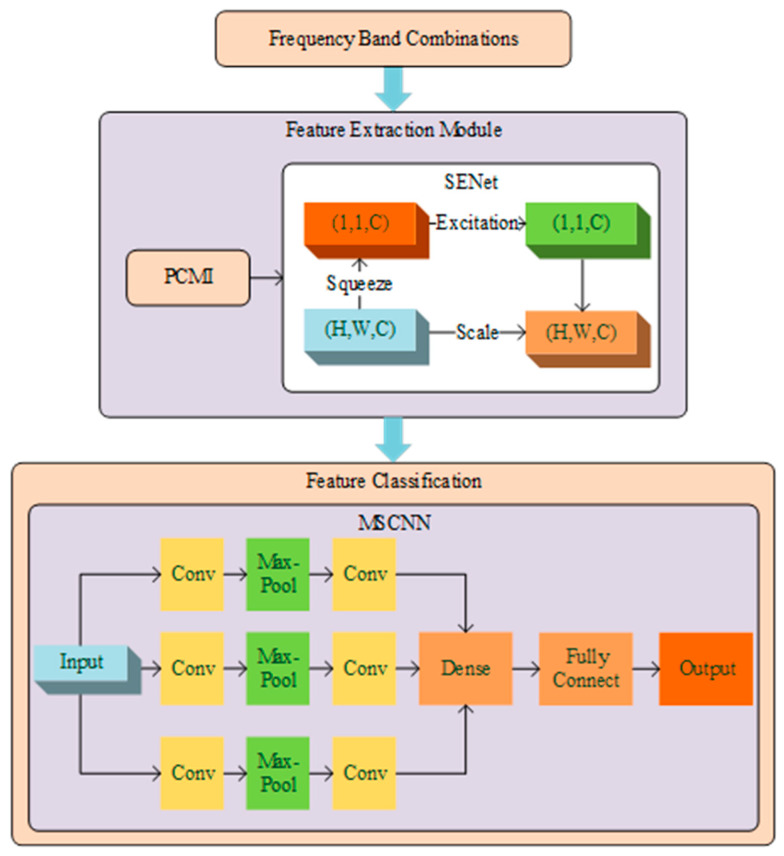
MSSECNN model.

Building upon SENet and MSCNN, this study proposes the MSSECNN to extract multi-scale information from input features and implement adaptive adjustments of global information through the squeezed excitation module. The structure of this network is shown in Figure 4. It consists of three modules: PCMI feature extraction, SENet module, and MSCNN feature classification.

First, the PCMI method extracts nonlinear spatial features from EEG signals, converting them into feature matrices across different frequency bands to capture inter-regional coupling information. Next, the SENet module applies adaptive weighting to these frequency band features, emphasizing critical frequency bands. Finally, the MSCNN module performs multi-scale convolution processing on the vital frequency band features, using convolution kernels of different sizes to extract both local and global features, enhancing the model’s adaptability to information at various scales.

This framework achieves efficient feature extraction and classification in EEG signal analysis through nonlinear feature extraction, multi-scale processing, and adaptive weighting, contributing to greater accuracy and practicality in spatial cognitive assessment.

**Figure 5:**
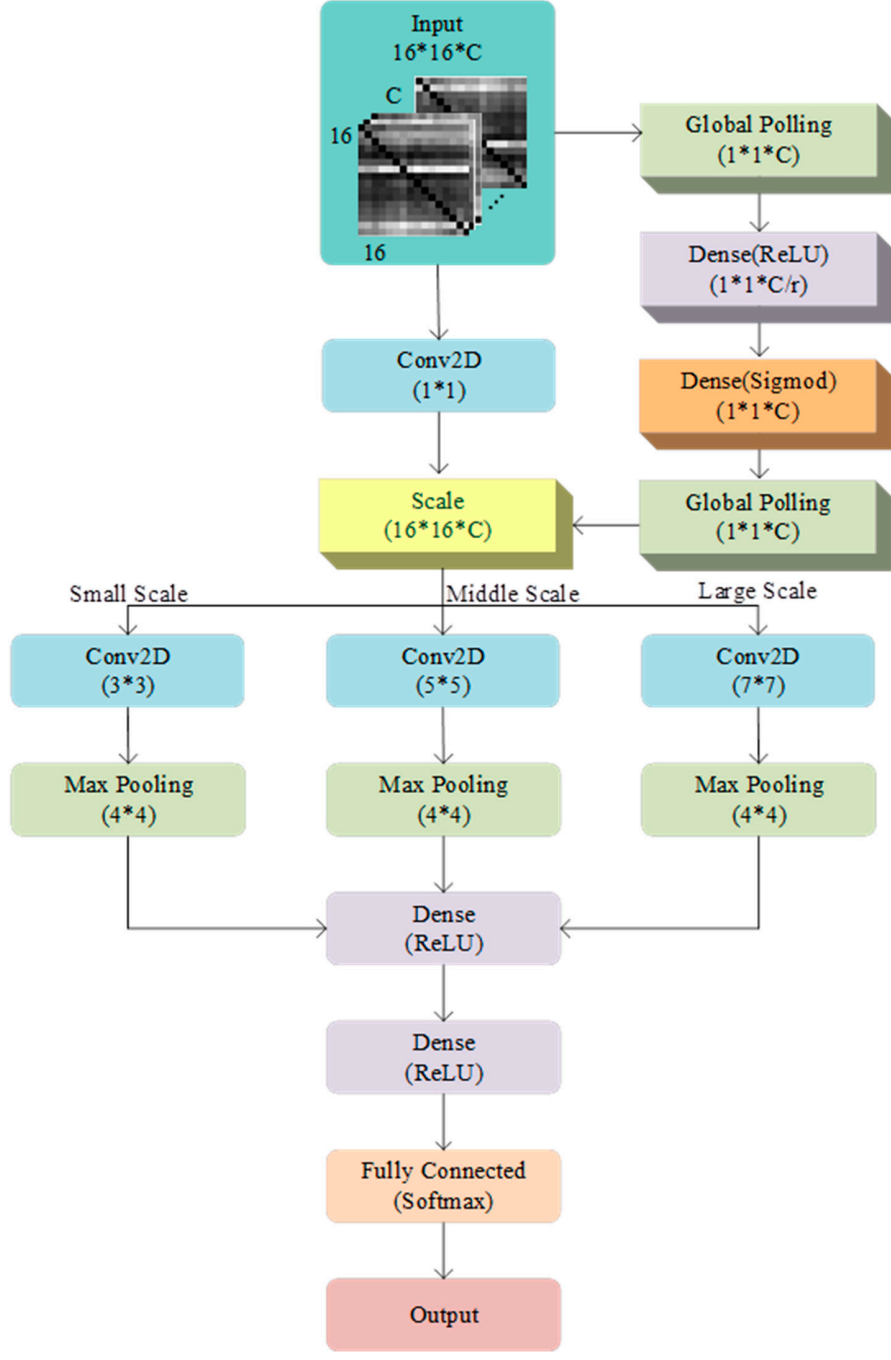
Multi-scale squeezed excitation network.

As shown in Figure 5, after processing with PCMI, the feature dimensions obtained are 16 × 16 × C, where C represents the number of frequency bands. This input feature first passes through a 1 × 1 convolution layer to adjust the number of frequency bands without changing the spatial dimensions, thereby preserving the essential spatial information of the input features.

The features processed by the 1 × 1 convolution are then directed to three parallel convolution branches. The first branch uses a 3 × 3 convolution kernel, outputting a two-channel feature map, followed by a 4 × 4 max pooling layer for downsampling. The second branch uses a 5 × 5 convolution kernel, outputting a two-channel feature map, also followed by 4 × 4 max pooling. The third branch uses a 7 × 7 convolution kernel, outputting a two-channel feature map with downsampling by 4 × 4 max pooling. This multi-scale convolution design enables the network to capture multi-scale information under different receptive fields, adapting to feature patterns of varying sizes and shapes.

After convolutional feature extraction, the features undergo channel-wise adaptive adjustment through the squeezed excitation module. First, global pooling is applied to each channel to obtain a global feature representation of size 1 × 1 × C. This global representation is passed through a transformation function composed of fully connected layers to generate adaptive weights for each channel. These layers learn the dependencies between channels, and the final layer uses Sigmoid activation to output weights in the range [0, 1], representing the relative importance of each channel. These weights are then used to modulate the original features, enabling the network to enhance relevant features and suppress irrelevant ones in a channel-wise manner.

The features processed by the squeezed excitation module are further integrated and processed through two fully connected layers. The first fully connected layer outputs 512 dimensions with ReLU activation, while the second layer outputs 256 dimensions with ReLU activation. Finally, the features from the two fully connected layers are fed into another two fully connected layers with Softmax activation for the final classification result.

This multi-scale squeezed excitation network combines convolutional and squeezed excitation modules to achieve multi-scale spatial information extraction and adaptive channel-wise adjustment, enhancing the network’s capacity to model complex features. This network accurately captures the spatial and channel dependencies of features in classification tasks, thereby enhancing classification performance.

### 3.5. Model Training and Evaluation Metrics

This study uses the TensorFlow deep learning framework to classify EEG data from spatial cognitive training before and after training. All models were split using five-fold cross-validation to ensure generalization capability. The training batch size for each model was set to 64, with 250 iterations. All models used the Adam optimizer with an initial learning rate of 0.0001. A learning rate scheduler was incorporated in the MSCNN and MSSECNN to gradually decay the learning rate, facilitating stable convergence and improving final classification performance.

Standard classification metrics were used to evaluate model performance, including precision, recall, F1-score, and area under curve (AUC). Classification accuracy and loss curves were also employed to assess model performance. Precision and recall were selected as the primary metrics to validate the performance of the MSCNN in classifying EEG feature data.

## 4. Results

The proposed model was evaluated on its ability to classify EEG signals into two distinct categories: pre-training cognitive state and post-training cognitive state. To validate the effectiveness of the proposed MSSECNN, this research compared traditional CNN, SECNN, and MSCNN models. In this EEG signal classification task, the CNN performs direct classification on PCMI-extracted signal features, while the SECNN incorporates SENet into the CNN, enhancing the classification by weighing key channel features. The MSCNN builds on the CNN by integrating a multi-scale convolution network, using convolutional kernels of various sizes to enhance multi-scale feature extraction of PCMI features. The MSSECNN combines multi-scale convolution and squeezed excitation modules, leveraging multi-scale feature extraction and adaptive weighting to improve feature representation and achieve superior classification performance.

The experimental dataset includes PCMI-feature EEG signals from the brain-controlled car and hand-controlled UAV groups. The performance of this model was evaluated across multiple frequency band combinations, specifically Delta–Theta–Alpha1–Alpha2–Beta1–Beta2–Gamma, Delta–Theta–Alpha2–Beta1–Beta2–Gamma, Delta–Alpha2–Beta1–Beta2–Gamma, Delta–Alpha2–Beta2–Gamma, Delta–Alpha2–Gamma, and Delta–Gamma.

The algorithms were executed in the hardware environment detailed in Table 1, ensuring consistent and reliable performance across all models.

### 4.1. Brain-Controlled Car Dataset

(1) Delta–Theta–Alpha1–Alpha2–Beta1–Beta2–Gamma

**Figure 6:**
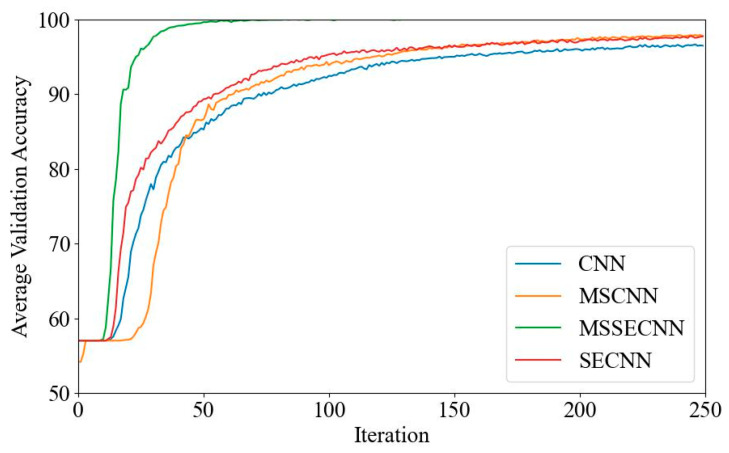
Average validation accuracy curve.

**Figure 7:**
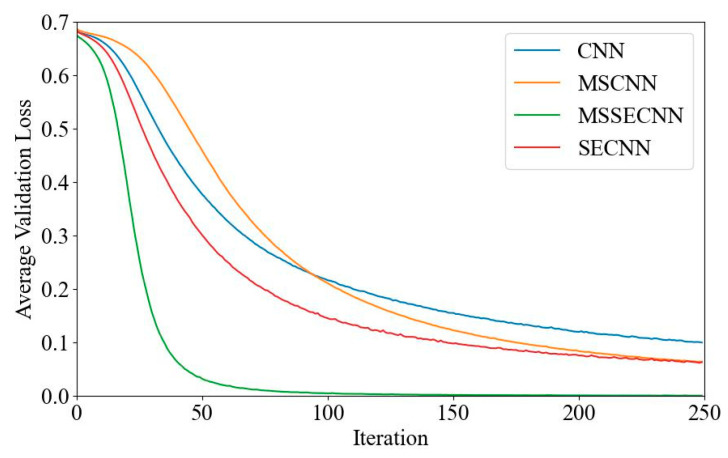
Average validation loss curve.

Figure 6 and Figure 7 present the average validation accuracy and loss curves for PCMI features across different models.

In Figure 6, the CNN model shows the lowest average validation accuracy, stabilizing around 96.5% after 180–200 iterations. The SECNN and MSCNN achieve higher accuracy, stabilizing around 97.5% after 160–180 iterations, while the MSSECNN achieves a stable validation accuracy of 100% within only 60–80 iterations.

Figure 7 shows the average validation loss curves for each model. The CNN model exhibits the highest validation loss, with a minimum loss value of around 0.1. The SECNN and MSCNN have lower minimum loss values, close to 0.06, while the MSSECNN achieves the lowest validation loss, approximately 0.0004.

Table 2 lists the average performance metrics for PCMI features across models. As shown, the MSSECNN outperforms all other models in every metric, achieving an accuracy of 100%. The SECNN and MSCNN show slightly lower accuracy, around 97.8%, while the CNN has the lowest accuracy at 96.57%.

(2) Delta–Theta–Alpha2–Beta1–Beta2–Gamma

**Figure 8:**
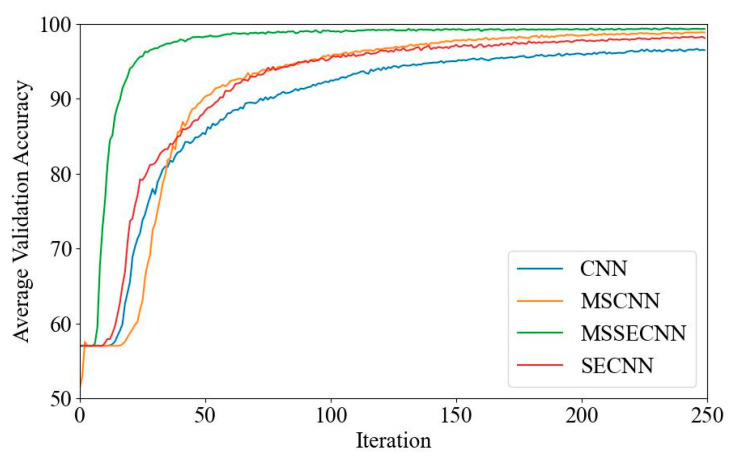
Average validation accuracy curve.

**Figure 9:**
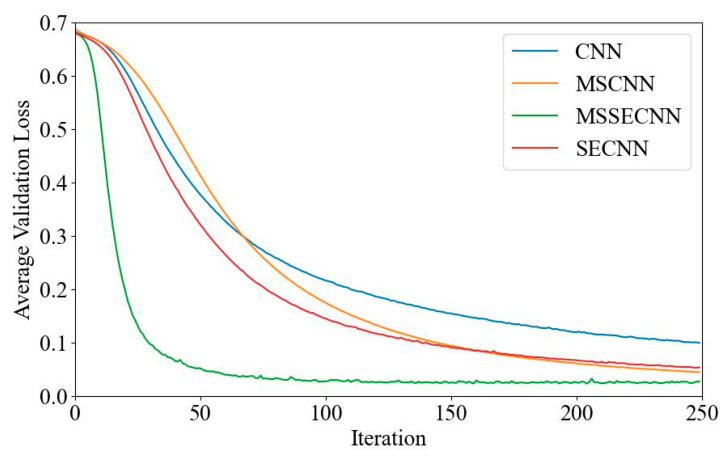
Average validation loss curve.

Figure 8 and Figure 9 show the average validation accuracy and loss curves for PCMI features in different models. In Figure 8, the CNN model has the lowest average validation accuracy, stabilizing at 95.9% after 180–200 iterations. The SECNN and MSCNN stabilize at around 98.2% accuracy, while the MSSECNN stabilizes at 99.35% within only 60–80 iterations.

Figure 9 shows the average validation loss curves, with the CNN exhibiting the highest loss, reaching a minimum of approximately 0.11. The SECNN and MSCNN have lower minimum losses, around 0.05, while the MSSECNN achieves the lowest minimum loss of approximately 0.026.

Table 3 lists the average evaluation metrics under this frequency band combination. The MSSECNN again outperforms all models, with an accuracy of 99.35%. The SECNN and MSCNN achieve around 98%, while the CNN has the lowest accuracy at 95.92%.

(3) Delta–Alpha2–Beta1–Beta2–Gamma

**Figure 10:**
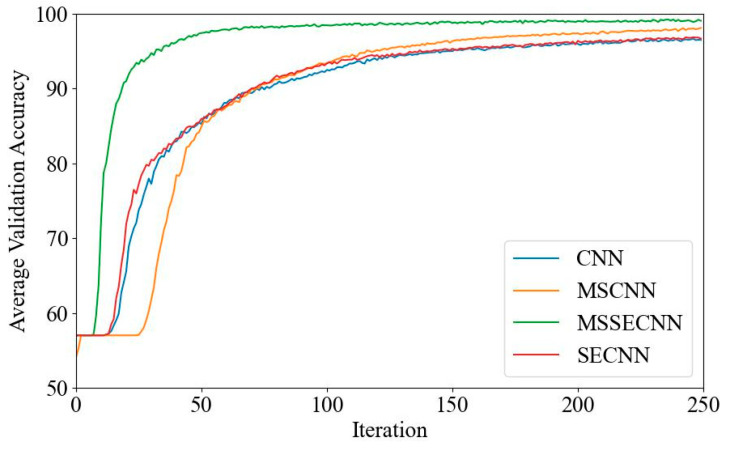
Average validation accuracy curve.

**Figure 11:**
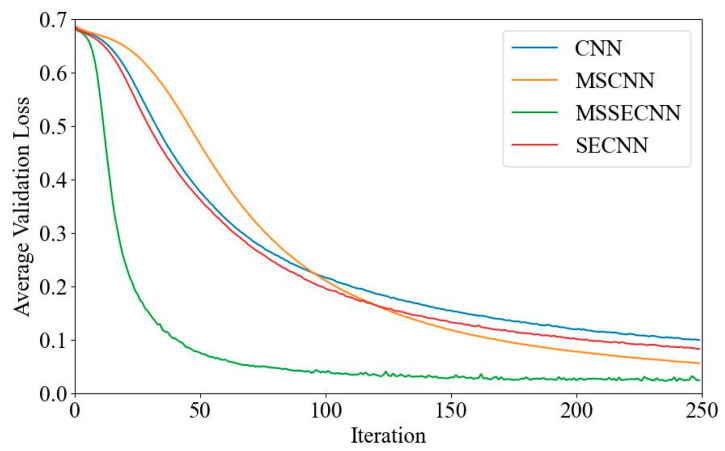
Average validation loss curve.

Figure 10 and Figure 11 present the average validation accuracy and loss curves for PCMI features across models. In Figure 10, the CNN and SECNN have relatively lower validation accuracy, stabilizing around 96% after 180–200 iterations. The MSCNN reaches a higher accuracy of approximately 98.1%, while the MSSECNN achieves stability at 99.1% within 60–80 iterations.

Figure 11 shows the average validation loss curves. The CNN model shows the highest validation loss, with a minimum loss of around 0.13. The SECNN and MSCNN achieve lower minimum loss values, approximately 0.08 and 0.06, respectively, while the MSSECNN has the lowest minimum loss, around 0.024.

Table 4 lists the average performance metrics for each model. The MSSECNN demonstrates the highest accuracy at 99.1%, with the SECNN and MSCNN achieving relatively high accuracy of 96.77% and 98.13%, respectively, while the CNN has the lowest accuracy at 95.46%.

(4) Delta–Alpha2–Beta2–Gamma

**Figure 12:**
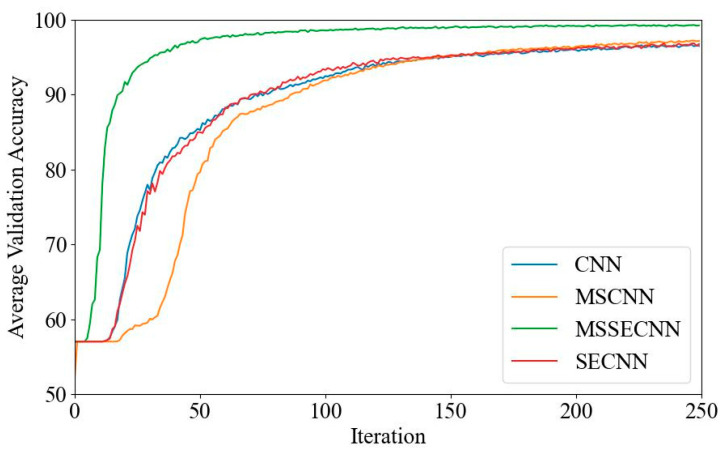
Average validation accuracy curve.

**Figure 13:**
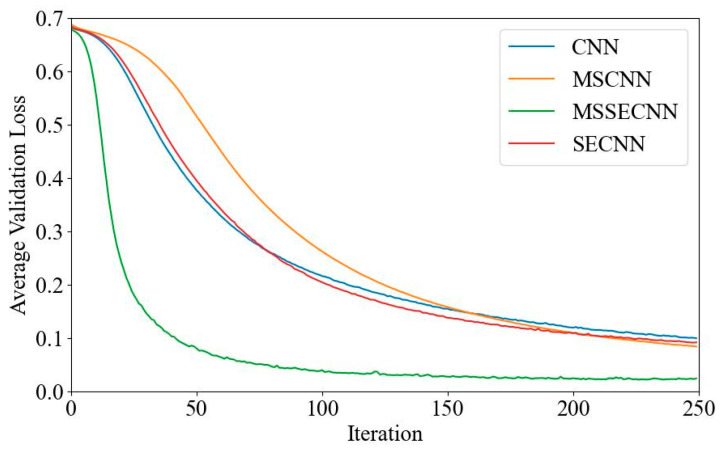
Average validation loss curve.

Figure 12 and Figure 13 present the average validation accuracy and loss curves for the CNN, SECNN, MSCNN, and MSSECNN on PCMI-based EEG features. In Figure 12, the CNN model shows the lowest validation accuracy, stabilizing at around 93.5% after 200–220 iterations. The SECNN and MSCNN reach higher accuracy, stabilizing at approximately 97% after 180–200 iterations, while the MSSECNN achieves the highest accuracy of 99.2% within 60–80 iterations.

Figure 13 shows the average validation loss curves. The CNN has the highest validation loss with a minimum of around 0.17, while the SECNN and MSCNN achieve lower minimum loss values, approximately 0.08. The MSSECNN has the lowest validation loss, close to 0.024.

Table 5 lists the evaluation metrics under this frequency band combination. The MSSECNN outperforms all other models, achieving an accuracy of 99.24%, while the SECNN and MSCNN achieve relatively high accuracy, around 97%. The CNN has the lowest accuracy at 93.47%.

(5) Delta–Alpha2–Gamma

**Figure 14:**
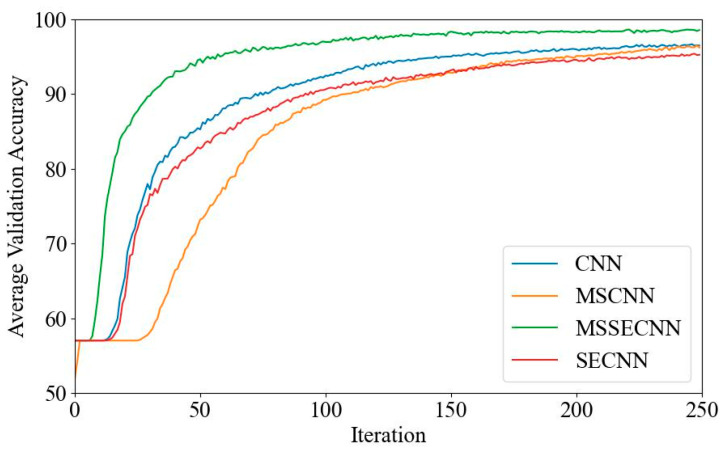
Average validation accuracy curve.

**Figure 15:**
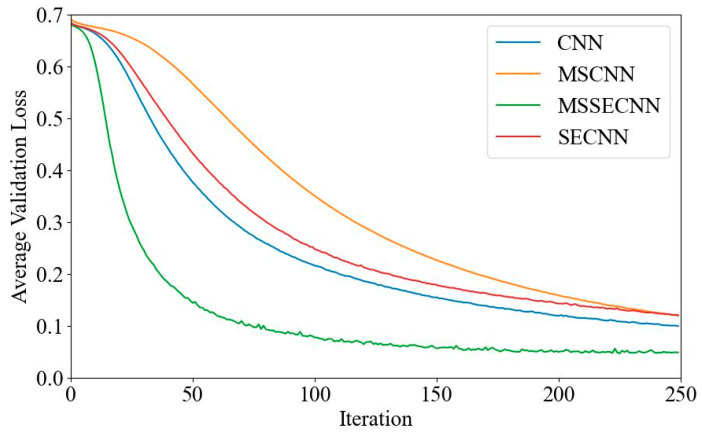
Average validation loss curve.

Figure 14 and Figure 15 present the average validation accuracy and loss curves for different models on PCMI-based EEG features. In Figure 14, the CNN shows the lowest validation accuracy, stabilizing at around 92.2% after 200–220 iterations. The SECNN and MSCNN achieve higher accuracy, stabilizing at approximately 96% after 180–200 iterations. The MSSECNN reaches the highest accuracy of 98.5% within 160–180 iterations.

Figure 15 shows the average validation loss curves. The CNN has the highest minimum validation loss, around 0.18, while the SECNN and MSCNN achieve lower minimum losses, around 0.12. The MSSECNN has the lowest minimum loss, approximately 0.048.

Table 6 lists the performance metrics for this frequency band combination. The MSSECNN achieves the highest scores across all metrics with an accuracy of 98.58%, while the SECNN and MSCNN perform slightly lower, around 96%. The CNN has the lowest accuracy at 92.23%.

(6) Delta–Gamma

**Figure 16:**
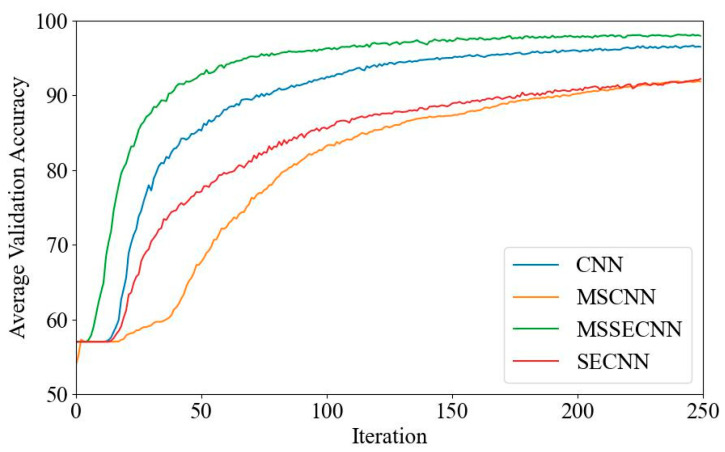
Average validation accuracy curve.

**Figure 17:**
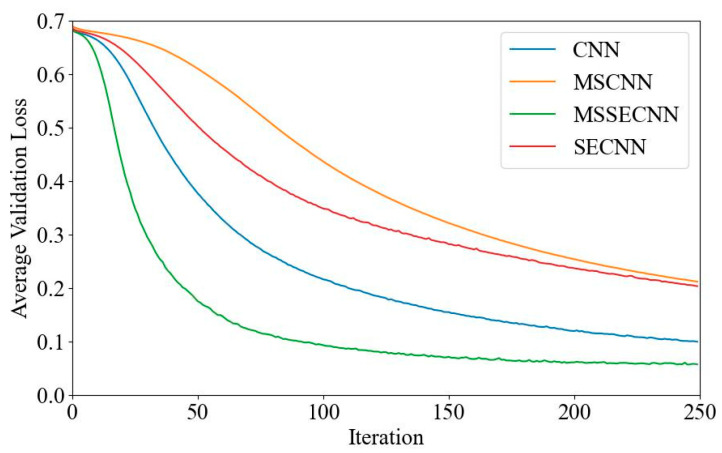
Average validation loss curve.

Figure 16 and Figure 17 present the average validation accuracy and loss curves for different models. In Figure 16, the CNN has the lowest validation accuracy, stabilizing at around 89.7% after 200–220 iterations. The SECNN and MSCNN achieve higher accuracy, around 92%, while the MSSECNN reaches the highest validation accuracy, stabilizing at 97.9% within 160–180 iterations.

Figure 17 shows the average validation loss curves. The CNN has the highest minimum validation loss, around 0.27, while the SECNN and MSCNN achieve lower minimum losses, around 0.20. The MSSECNN achieves the lowest validation loss, around 0.058.

Table 7 lists the evaluation metrics under this frequency band combination. The MSSECNN achieves the highest scores across all metrics with an accuracy of 97.99%, while the SECNN and MSCNN perform slightly lower, around 92%. The CNN has the lowest accuracy at 89.77%.

### 4.2. Hand-Controlled UAV Dataset

During cognitive tasks, hand movements, turns, and other motions of participants can introduce environmental n.

(1) Delta–Theta–Alpha1–Alpha2–Beta1–Beta2–Gamma

**Figure 18:**
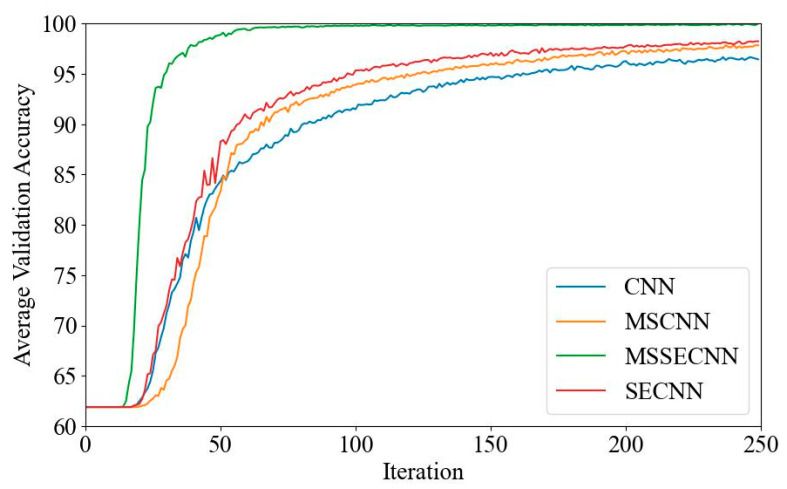
Average validation accuracy curve.

**Figure 19:**
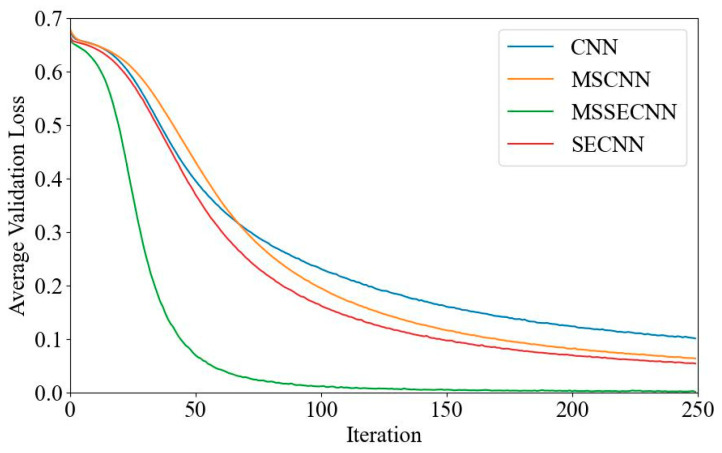
Average validation loss curve.

Figure 18 and Figure 19 present the average validation accuracy and loss curves for PCMI features across different models (CNN, SECNN, MSCNN, and MSSECNN). In Figure 18, the CNN model shows the lowest average validation accuracy, stabilizing at approximately 96.3% after 200–220 iterations. The SECNN and MSCNN achieve higher accuracy, stabilizing near 98% after 180–200 iterations, while the MSSECNN reaches stability with a validation accuracy of 99.9% within 60–80 iterations.

Figure 19 shows the average validation loss curves for each model. The CNN model shows the highest loss, with a minimum value of around 0.1. The SECNN and MSCNN have lower minimum loss values, approximately 0.06, while the MSSECNN achieves the lowest validation loss at close to 0.002.

Table 8 lists the evaluation metrics for each model under this frequency band combination. The MSSECNN outperforms all models with an accuracy of 99.97%, while the SECNN and MSCNN achieve close to 98% accuracy. The CNN exhibits the lowest accuracy at 96.31%.

(2) Delta–Theta–Alpha2–Beta1–Beta2–Gamma

**Figure 20:**
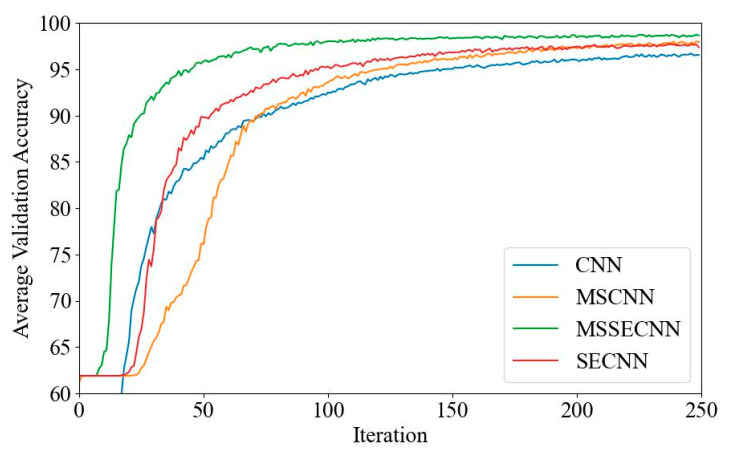
Average validation accuracy curve.

**Figure 21:**
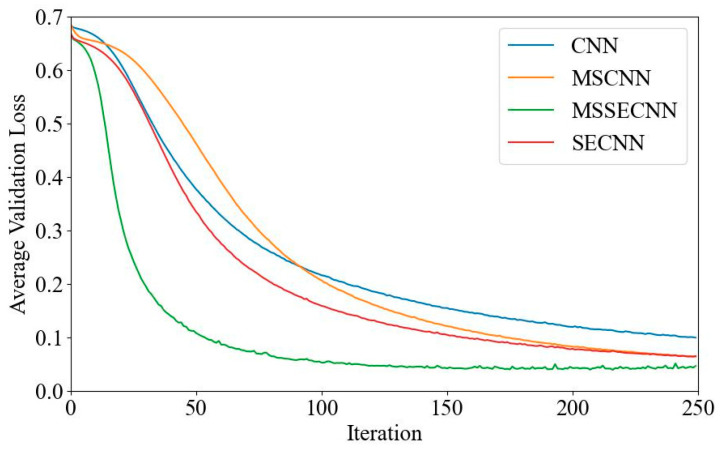
Average validation loss curve.

Figure 20 and Figure 21 present the average validation accuracy and loss curves for PCMI features across different models. In Figure 20, the CNN model stabilizes at a relatively low accuracy of 96% after 200–220 iterations. The SECNN and MSCNN stabilize around 97% accuracy after 180–200 iterations, while the MSSECNN reaches 98.6% within 60–80 iterations.

Figure 21 shows the average validation loss curves. The CNN model shows the highest loss, with a minimum value of about 0.098. The SECNN and MSCNN reach minimum loss values around 0.065, while the MSSECNN achieves the lowest validation loss, at approximately 0.044.

Table 9 lists the evaluation metrics for this frequency band combination. The MSSECNN outperforms all models with an accuracy of 98.62%, while the SECNN and MSCNN achieve close to 97% accuracy. The CNN exhibits the lowest accuracy at 96.08%.

(3) Delta–Alpha2–Beta1–Beta2–Gamma

**Figure 22:**
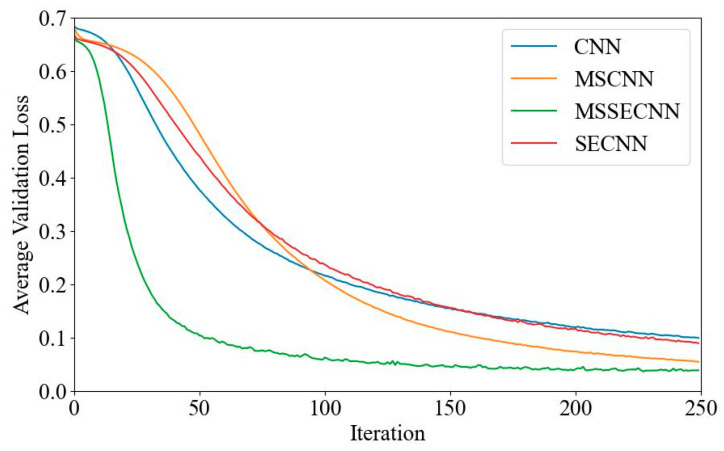
Average validation accuracy curve.

**Figure 23:**
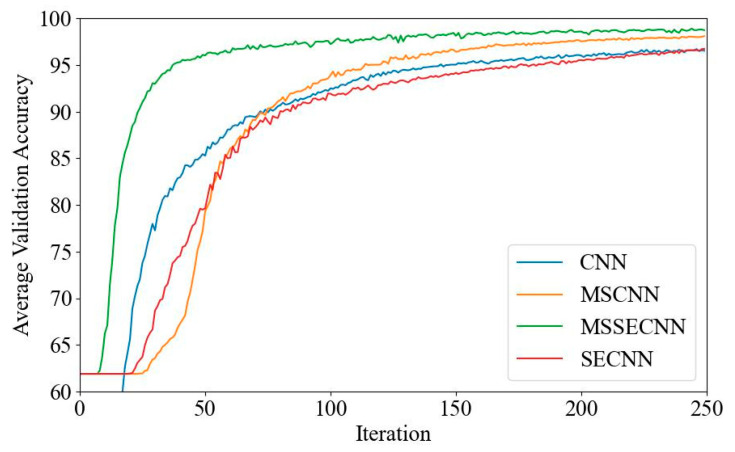
Average validation loss curve.

Figure 22 and Figure 23 present the average validation accuracy and loss curves for PCMI features across different models. In Figure 22, theCNN and SECNN achieve relatively low validation accuracy, stabilizing around 96% after 200–220 iterations. The MSCNN achieves higher accuracy, stabilizing at 97.9% after 180–200 iterations, while the MSSECNN achieves 98.6% accuracy within 60–80 iterations.

Figure 23 shows the average validation loss curves. The CNN and SECNN show higher loss values, with minimum losses of around 0.1. The MSCNN achieves a lower minimum loss of approximately 0.055, while the MSSECNN reaches the lowest minimum loss at close to 0.038.

Table 10 lists the evaluation metrics for this frequency band combination. The MSSECNN outperforms all models with an accuracy of 98.65%, while the MSCNN achieves a slightly lower accuracy of 97.94%. The CNN and SECNN achieve the lowest accuracy, around 96%.

(4) Delta–Alpha2–Beta2–Gamma

**Figure 24:**
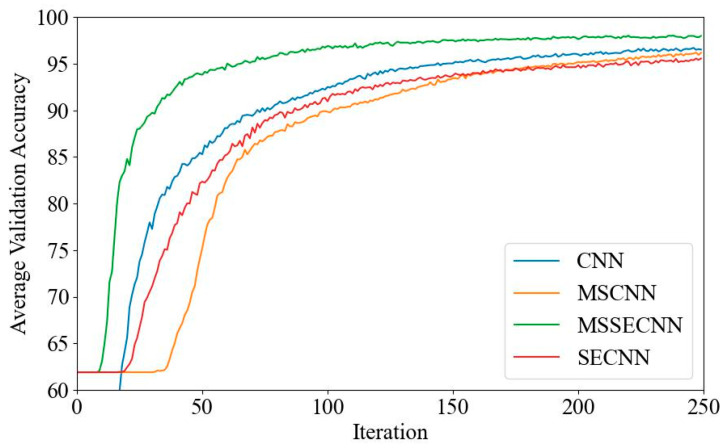
Average validation accuracy curve.

**Figure 25:**
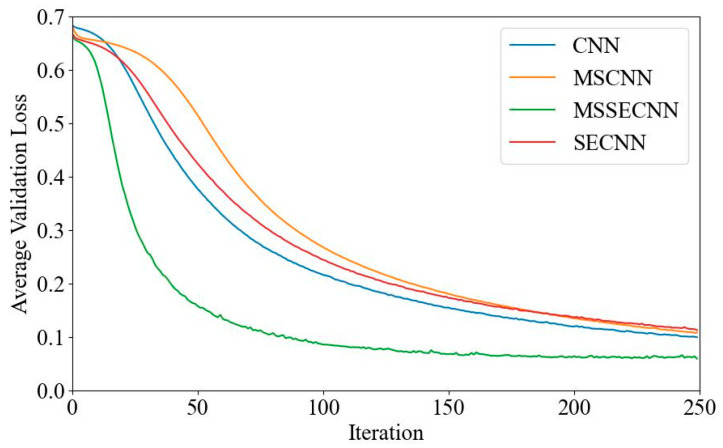
Average validation loss curve.

Figure 24 and Figure 25 present the average validation accuracy and loss curves for the CNN, SECNN, MSCNN, and MSSECNN on PCMI-based EEG features. In Figure 24, the CNN model has the lowest validation accuracy, stabilizing around 93.8% after 200–220 iterations. The SECNN and MSCNN reach higher accuracy, stabilizing around 95% after 180–200 iterations. The MSSECNN achieves the highest validation accuracy of 97.9% within 160–180 iterations.

Figure 25 shows the average validation loss curves. The CNN has the highest minimum validation loss, around 0.156, while the SECNN and MSCNN achieve lower minimum loss values, around 0.1. The MSSECNN reaches the lowest validation loss at approximately 0.062.

Table 11 lists the evaluation metrics for this frequency band combination. The MSSECNN outperforms all models, achieving an accuracy of 97.99%. The SECNN and MSCNN are closely followed with around 95% accuracy, while the CNN has the lowest accuracy at 93.8%.

(5) Delta–Alpha2–Gamma

**Figure 26:**
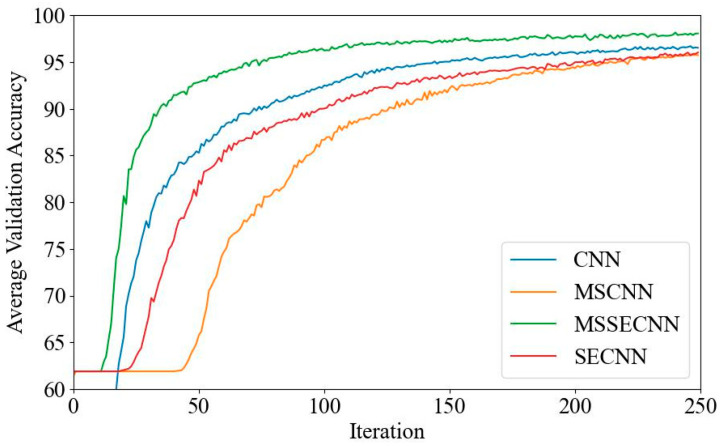
Average validation accuracy curve.

**Figure 27:**
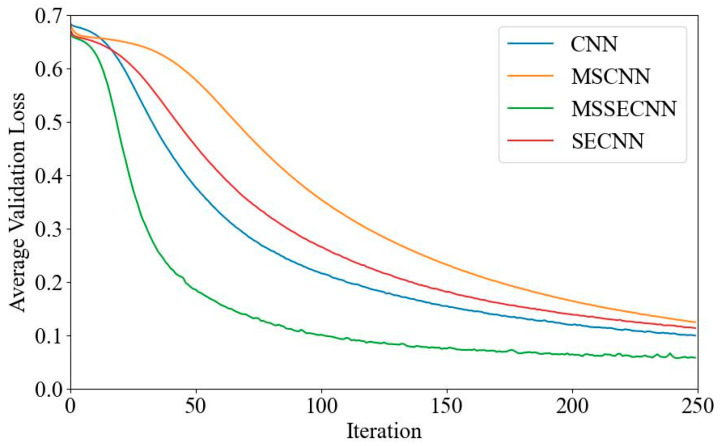
Average validation loss curve.

Figure 26 and Figure 27 present the average validation accuracy and loss curves for PCMI features across models. In Figure 26, the CNN model shows the lowest validation accuracy, stabilizing around 92.3% after 200–220 iterations. The SECNN and MSCNN achieve higher accuracy, stabilizing around 95% after 180–200 iterations. The MSSECNN achieves the highest accuracy of 98% within 160–180 iterations.

Figure 27 shows the average validation loss curves. The CNN shows the highest validation loss with a minimum of around 0.185, while the SECNN and MSCNN reach lower minimum losses of around 0.12. The MSSECNN has the lowest minimum loss, close to 0.058.

Table 12 lists the evaluation metrics for this frequency band combination. The MSSECNN achieves the highest accuracy of 98.02%, while the SECNN and MSCNN perform well with close to 95% accuracy. The CNN has the lowest accuracy at 92.32%.

(6) Delta–Gamma

**Figure 28:**
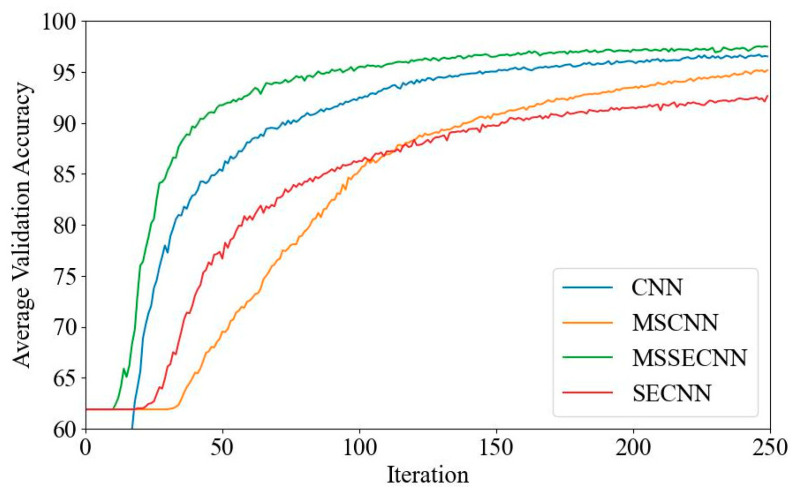
Average validation accuracy curve.

**Figure 29:**
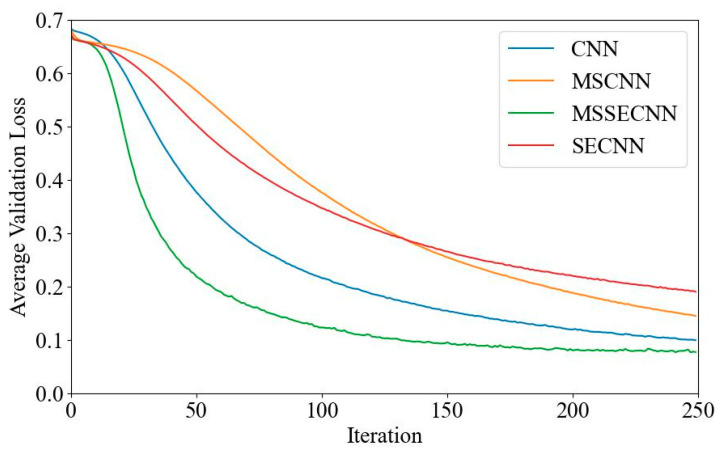
Average validation loss curve.

Figure 28 and Figure 29 present the average validation accuracy and loss curves for different models. In Figure 28, the CNN and SECNN achieve lower validation accuracy, stabilizing around 91% after 200–220 iterations. The MSCNN reaches higher accuracy, stabilizing at 95.1% after 180–200 iterations. The MSSECNN achieves the highest accuracy of 97.4% within 160–180 iterations.

Figure 29 shows the average validation loss curves. The CNN and SECNN show relatively high losses, with minimum losses of around 0.20. The MSCNN achieves a lower minimum loss of approximately 0.148, while the MSSECNN reaches the lowest minimum loss, around 0.08.

Table 13 lists the evaluation metrics for this frequency band combination. The MSSECNN achieves the highest accuracy of 97.37%, while the MSCNN follows closely with 95.12% accuracy. The CNN and SECNN have the lowest accuracy, both around 91%.

## 5. Discussion

### 5.1. Advantages of the MSSECNN Architecture

In EEG signal analysis, CNNs are widely used as foundational deep learning models for EEG classification due to their effectiveness in spatial feature extraction [39,55]. However, traditional EEG analysis methods frequently lack the capability to capture the comprehensive multi-scale and nonlinear features inherent in EEG signals. The fixed convolution kernel size in CNNs makes it challenging to capture feature variations across different frequency bands in EEG signals, and they need more adaptive weighting for feature channels, leading to suboptimal classification accuracy and robustness [56].

Researchers introduced the MSCNN to capture multi-level features using convolutional kernels of various sizes, accommodating the multi-scale nature of signals [42]. While the MSCNN can effectively capture features across scales, it lacks channel differentiation, making it challenging to emphasize the most distinctive frequency band features in EEG signals [57].

In this study, we propose the MSSECNN, which combines SENet with multi-scale convolution. The adaptive weighting mechanism of SENet assigns different weights to each channel, effectively emphasizing key channels and enhancing feature selection during classification [58]. By combining multi-scale convolution with squeeze-and-excitation modules, the MSSECNN effectively extracts complex EEG features across various scales and adaptively emphasizes crucial frequency bands, resulting in enhanced classification accuracy and robustness.

Experimental results indicate that the MSSECNN achieves higher validation accuracy and stability with fewer iterations than traditional CNN, SECNN, and MSCNN models. The integration of multi-scale convolution and adaptive weighting in the MSSECNN highlights its strong capability in classifying complex EEG signals, emphasizing its effectiveness and potential for EEG analysis.

Despite the high accuracy achieved by the MSSECNN model, several limitations remain. First, the current study focuses on binary classification and does not explore more complex multi-class or multi-task classification scenarios. Additionally, while the model demonstrates strong performance on the collected dataset, further investigation is needed to validate its robustness on larger, multi-center datasets with greater variability in EEG signals.

Future works will focus on enhancing the adaptability of the proposed model for real-world applications. This includes investigating advanced deep learning architectures, such as Transformer-based models or hybrid frameworks combining CNNs and recurrent neural networks, to better capture both spatial and temporal dependencies in EEG signals. Furthermore, integrating multi-modal EEG data, including functional near-infrared spectroscopy (fNIRS) or magnetoencephalography (MEG), could provide complementary information to improve classification accuracy. Real-time EEG signal processing and classification also represent critical areas for further development, particularly for applications in brain-computer interfaces and cognitive monitoring systems.

### 5.2. Feature Capacity of Multi-Frequency Band Combinations

In EEG signal analysis, single-frequency band approaches can capture certain cognitive features but struggle to fully represent the brain’s multi-level activity in complex cognitive tasks [59]. Different EEG frequency bands correspond to distinct cognitive functions, making single-band analysis inadequate for handling diverse and complex tasks, particularly in spatial cognition [60].

Researchers have explored multi-frequency band combinations to capture more comprehensive cognitive information, integrating features from multiple frequency bands to enhance EEG classification accuracy and robustness [61,62]. These multi-frequency band combinations offer synergistic insights across bands, better representing overall brain activity [63]. However, multi-frequency band combinations also introduce feature redundancy, and selecting and combining appropriate frequency bands significantly impacts classification performance [64,65].

Based on the results of this study, the MSSECNN achieved the best classification performance on the seven-band combined data, covering critical frequency ranges from low to high frequencies to reflect various brain activity levels comprehensively. Specifically, Delta and Theta bands are associated with attention and memory processes [66,67,68], Alpha1 and Alpha2 bands relate to perception and response coordination [69,70], Beta1 and Beta2 bands play critical roles in task execution and inter-regional brain interactions [71], and the Gamma band is closely linked to higher cognitive functions [72,73]. This frequency band combination enables the MSSECNN to capture multi-level cognitive features during spatial cognitive tasks, improving classification accuracy and robustness.

Experimental results show that the seven-frequency band MSSECNN significantly outperforms other models and frequency combinations regarding classification accuracy, recall, F1-score, and AUC, particularly in complex tasks. The superiority of this combination substantiates the complementary nature of multi-band features, reinforcing its efficacy for EEG analysis. It highlights the importance of multi-frequency band combinations in EEG analysis, offering a reference for future multi-frequency band research in EEG signal analysis.

## 6. Conclusions

Compared with traditional networks such as the CNN, SECNN, and MSCNN, the proposed MSSECNN model demonstrates significant classification accuracy, robustness, and feature extraction capability advantages. Particularly in multi-frequency band combinations, the multi-scale convolution and adaptive weighting mechanisms of the MSSECNN effectively capture complex EEG signal features, making it highly suitable for spatial cognitive tasks and providing a more precise solution for EEG feature extraction and classification.

## Figures and Tables

**Table 1:** Hardware environment.

Hardware Component	Specifications
Processor	Intel^®^ Core™ i5-10400F CPU @ 2.90 GHz (Intel Corporation, Santa Clara, CA, USA)
GPU	NVIDIA GeForce RTX 2060 (NVIDIA Corporation, Santa Clara, CA, USA)
Memory	16 GB DDR4
Storage	1 TB SSD
Operating System	Windows 10 (Microsoft Corporation, Redmond, WA, USA)

**Table 2:** Performance metrics for PCMI features across models.

Classifier	Precision	Recall	F1-Score	AUC
CNN	98.26%	93.38%	95.76%	0.990
SECNN	97.52%	97.52%	97.52%	0.993
MSCNN	98.77%	99.58%	99.17%	0.993
MSSECNN	99.17%	99.58%	99.38%	0.995

**Table 3:** Performance metrics for PCMI features across models.

Classifier	Precision	Recall	F1-Score	AUC
CNN	94.35%	96.69%	95.51%	0.989
SECNN	97.04%	95.04%	96.03%	0.993
MSCNN	93.87%	95.04%	94.45%	0.994
MSSECNN	99.58%	98.76%	99.17%	0.995

**Table 4:** Performance metrics for PCMI features across models.

Classifier	Precision	Recall	F1-Score	AUC
CNN	95.79%	94.21%	95.00%	0.985
SECNN	97.14%	98.34%	97.74%	0.992
MSCNN	97.54%	98.34%	97.94%	0.993
MSSECNN	100%	99.17%	99.58%	0.995

**Table 5:** Performance metrics for PCMI features across models.

Classifier	Precision	Recall	F1-Score	AUC
CNN	95.00%	94.21%	94.60%	0.978
SECNN	92.06%	95.86%	93.92%	0.990
MSCNN	91.09%	92.97%	92.02%	0.991
MSSECNN	100%	99.17%	99.58%	0.995

**Table 6:** Performance metrics for PCMI features across models.

Classifier	Precision	Recall	F1-Score	AUC
CNN	93.96%	90.08%	91.98%	0.974
SECNN	95.70%	92.14%	93.89%	0.987
MSCNN	88.01%	97.10%	92.33%	0.989
MSSECNN	99.17%	99.58%	99.38%	0.994

**Table 7:** Performance metrics for PCMI features across models.

Classifier	Precision	Recall	F1-Score	AUC
CNN	88.98%	86.77%	87.86%	0.952
SECNN	94.06%	91.73%	92.88%	0.973
MSCNN	94.40%	97.52%	95.93%	0.971
MSSECNN	99.16%	98.34%	98.75%	0.994

**Table 8:** Performance metrics for PCMI features across models.

Classifier	Precision	Recall	F1-Score	AUC
CNN	92.72%	95.32%	94.00%	0.989
SECNN	93.75%	98.59%	96.10%	0.993
MSCNN	95.83%	97.18%	96.50%	0.993
MSSECNN	99.06%	99.06%	99.06%	0.995

**Table 9:** Performance metrics for PCMI features across models.

Classifier	Precision	Recall	F1-Score	AUC
CNN	88.88%	97.19%	92.85%	0.990
SECNN	92.57%	99.53%	95.92%	0.993
MSCNN	97.63%	96.71%	97.16%	0.993
MSSECNN	97.65%	97.65%	97.65%	0.994

**Table 10:** Performance metrics for PCMI features across models.

Classifier	Precision	Recall	F1-Score	AUC
CNN	91.30%	98.13%	94.59%	0.989
SECNN	93.66%	97.18%	95.39%	0.991
MSCNN	95.75%	95.30%	95.52%	0.993
MSSECNN	99.06%	99.53%	99.29%	0.994

**Table 11:** Performance metrics for PCMI features across models.

Classifier	Precision	Recall	F1-Score	AUC
CNN	92.52%	92.52%	92.52%	0.983
SECNN	93.72%	98.12%	95.87%	0.988
MSCNN	94.54%	97.65%	96.07%	0.989
MSSECNN	99.52%	98.59%	99.05%	0.993

**Table 12:** Performance metrics for PCMI features across models.

Classifier	Precision	Recall	F1-Score	AUC
CNN	84.87%	94.39%	89.38%	0.974
SECNN	90.39%	97.18%	93.66%	0.989
MSCNN	85.77%	93.42%	89.43%	0.988
MSSECNN	94.95%	97.18%	96.05%	0.994

**Table 13:** Performance metrics for PCMI features across models.

Classifier	Precision	Recall	F1-Score	AUC
CNN	92.15%	87.85%	89.95%	0.969
SECNN	82.44%	94.83%	88.20%	0.975
MSCNN	81.41%	96.38%	83.82%	0.985
MSSECNN	95.81%	96.71%	96.26%	0.992

## Data Availability

The datasets presented in this article are not readily available due to restrictions related to ongoing analyses and further research development. For any inquiries, please contact the corresponding author.

## References

[1] Ata A., Yeşılkaya B., Cura Ö.K., Akan A. Control of Serious Games Designed for Alzheimer’s and Dementia Patients by EEG Signals. Proceedings of the 2019 Medical Technologies Congress (TIPTEKNO).

[2] Vlček K., Laczó J. (2014). Neural correlates of spatial navigation changes in mild cognitive impairment and Alzheimer’s disease. Front. Behav. Neurosci..

[3] Huang Y., Xu J., Zhang X., Liu Y., Yu E. (2023). Research progress on vestibular dysfunction and visual–spatial cognition in patients with Alzheimer’s disease. Front. Aging Neurosci..

[4] Schneider C.B., Linse K., Schönfeld R., Brown S., Koch R., Reichmann H., Leplow B., Storch A. (2017). Spatial learning deficits in Parkinson’s disease with and without mild cognitive impairment. Park. Relat. Disord..

[5] Weisman D., McKeith I. (2007). Dementia with Lewy bodies. Seminars in Neurology.

[6] Meghdadi A.H., Stevanović Karić M., McConnell M., Rupp G., Richard C., Hamilton J., Salat D., Berka C. (2021). Resting state EEG biomarkers of cognitive decline associated with Alzheimer’s disease and mild cognitive impairment. PLoS ONE.

[7] Labidi J., Warniez A., Derambure P., Lebouvier T., Pasquier F., Delval A., Betrouni N. (2024). Qualitative versus quantitative assessment of electroencephalography in cognitive decline: Comparison in a clinical population. Neurophysiol. Clin..

[8] Zawiślak-Fornagiel K., Ledwoń D., Bugdol M., Grażyńska A., Ślot M., Tabaka-Pradela J., Bieniek I., Siuda J. (2024). Quantitative EEG Spectral and Connectivity Analysis for Cognitive Decline in Amnestic Mild Cognitive Impairment. J. Alzheimer’s Dis..

[9] Liu H., Wang J., Xin X., Wang P., Jiang W., Meng T. (2024). The relationship and pathways between resting-state EEG, physical function, and cognitive function in older adults. BMC Geriatr..

[10] Zandbagleh A., Miltiadous A., Sanei S., Azami H. (2024). Beta-to-Theta Entropy Ratio of EEG in Aging, Frontotemporal Dementia, and Alzheimer’s Dementia. Am. J. Geriatr. Psychiatry.

[11] Bureš J., Lánský P. (2004). From spreading depression to spatial cognition. Physiol. Res..

[12] Chiu T.C., Gramann K., Ko L.W., Duann J.R., Jung T.P., Lin C.T. (2012). Alpha modulation in parietal and retrosplenial cortex correlates with navigation performance. Psychophysiology.

[13] Chouinard S., Brière M.-È., Rainville C., Godbout R. (2003). Correlation between evening and morning waking EEG and spatial orientation. Brain Cogn..

[14] Nishiyama N., Mizuhara H., Miwakeichi F., Yamaguchi Y. Theta episodes observed in human scalp EEG during virtual navigation-spatial distribution and task dependence. Proceedings of the 9th International Conference on Neural Information Processing ICONIP’02.

[15] Lin C.-T., Chiu T.-C., Gramann K. (2015). EEG correlates of spatial orientation in the human retrosplenial complex. NeuroImage.

[16] Park J., Lee H., Kim T., Park G.Y., Lee E.M., Baek S., Ku J., Kim I.Y., Kim S.I., Jang D.P. (2014). Role of low-and high-frequency oscillations in the human hippocampus for encoding environmental novelty during a spatial navigation task. Hippocampus.

[17] White D.J., Congedo M., Ciorciari J., Silberstein R.B. (2012). Brain oscillatory activity during spatial navigation: Theta and gamma activity link medial temporal and parietal regions. J. Cogn. Neurosci..

[18] Sharma G., Salam A.A., Chandra S., Singh V., Mittal A. Influence of spatial learning perspectives on navigation through virtual reality environment. Proceedings of the Brain Informatics and Health: International Conference, BIH 2016.

[19] Snider J., Ahmed O.J., Halgren E., Poizner H., Cash S.S. Human intracranial recordings during spatial exploration of a 3D virtual environment. Proceedings of the 2013 6th International IEEE/EMBS Conference on Neural Engineering (NER).

[20] Kober S.E., Kurzmann J., Neuper C. (2012). Cortical correlate of spatial presence in 2D and 3D interactive virtual reality: An EEG study. Int. J. Psychophysiol..

[21] Ramos-Loyo J., Sanchez-Loyo L. (2011). Gender differences in EEG coherent activity before and after training navigation skills in virtual environments. Hum. Physiol..

[22] Pacheco Estefan D., Sánchez-Fibla M., Duff A., Principe A., Rocamora R., Zhang H., Axmacher N., Verschure P.F. (2019). Coordinated representational reinstatement in the human hippocampus and lateral temporal cortex during episodic memory retrieval. Nat. Commun..

[23] Sharma G., Gramann K., Chandra S., Singh V., Mittal A.P. (2017). Brain connectivity during encoding and retrieval of spatial information: Individual differences in navigation skills. Brain Inform..

[24] Li X., Yan Y., Wei W. (2013). Identifying Patients with Poststroke Mild Cognitive Impairment by Pattern Recognition of Working Memory Load-Related ERP. Comput. Math. Methods Med..

[25] Pergher V., Wittevrongel B., Tournoy J., Schoenmakers B., Van Hulle M.M. (2018). N-back training and transfer effects revealed by behavioral responses and EEG. Brain Behav..

[26] Baker T.E., Holroyd C.B. (2013). The topographical N170: Electrophysiological evidence of a neural mechanism for human spatial navigation. Biol. Psychol..

[27] Protopapa F., Mylonas D., Spiliotis K., Siettos C., Smyrnis N., Evdokimidis I. Dynamic analysis of EEG signals during spatial working memory used for either perception discrimination or planning of action. Proceedings of the 2011 Annual International Conference of the IEEE Engineering in Medicine and Biology Society.

[28] Toppi J., Astolfi L., Risetti M., Anzolin A., Kober S.E., Wood G., Mattia D. (2018). Different topological properties of EEG-derived networks describe working memory phases as revealed by graph theoretical analysis. Front. Hum. Neurosci..

[29] Gao H., Sheng R., Chen Z., Liu H., Xu S., Zhang B. (2024). Multi-scale Random-shape Convolution and Adaptive Graph Convolution Fusion Network for Hyperspectral Image Classification. IEEE Trans. Geosci. Remote Sens..

[30] Li X., Yang Z., Tu X., Wang J., Huang J. (2024). MFRC-Net: Multi-Scale Feature Residual Convolutional Neural Network for Motor Imagery Decoding. IEEE J. Biomed. Health Inform..

[31] Luo J., Cheng Q., Wang H., Du Q., Wang Y., Li Y. (2024). MI-MBFT: Superior Motor Imagery Decoding of Raw EEG Data Based on a Multi-Branch and Fusion Transformer Framework. IEEE Sens. J..

[32] Wang J., Zhang L., Hu B. (2012). Research on the methods for multi-class kernel CSP-based feature extraction. Sheng Wu Yi Xue Gong Cheng Xue Za Zhi J. Biomed. Eng. Shengwu Yixue Gongchengxue Zazhi.

[33] Hengzhi L., Dong W., Zhenhao W., Yanhong Z. (2019). Advances in the extraction and classification of EEG dynamic features in patients with mild cognitive impairment. Chin. J. Biomed. Eng..

[34] Li Y., Li X., Ouyang G., Guan X. (2007). Information flow among neural networks with Bayesian estimation. Chin. Sci. Bull..

[35] Li X., Ouyang G. (2010). Estimating coupling direction between neuronal populations with permutation conditional mutual information. Neuroimage.

[36] Wen D., Bian Z., Li Q., Wang L., Lu C., Li X. (2016). Resting-state EEG coupling analysis of amnestic mild cognitive impairment with type 2 diabetes mellitus by using permutation conditional mutual information. Clin. Neurophysiol..

[37] Wen D., Liang B., Li J., Wu L., Wan X., Dong X., Lan X., Song H., Zhou Y. (2023). Feature Extraction Method of EEG Signals Evaluating Spatial Cognition of Community Elderly with Permutation Conditional Mutual Information Common Space Model. IEEE Trans. Neural Syst. Rehabil. Eng..

[38] Wen D., Li R., Jiang M., Li J., Liu Y., Dong X., Saripan M.I., Song H., Han W., Zhou Y. (2022). Multi-dimensional conditional mutual information with application on the EEG signal analysis for spatial cognitive ability evaluation. Neural Netw..

[39] Mao W., Fathurrahman H., Lee Y., Chang T. (2020). EEG dataset classification using CNN method. Journal of Physics: Conference Series.

[40] Bird J.J., Faria D.R., Manso L.J., Ayrosa P.P., Ekart A. (2021). A study on CNN image classification of EEG signals represented in 2D and 3D. J. Neural Eng..

[41] Kundu S., Ari S. (2019). MsCNN: A deep learning framework for P300-based brain–computer interface speller. IEEE Trans. Med. Robot. Bionics.

[42] Roy A.M. (2022). An efficient multi-scale CNN model with intrinsic feature integration for motor imagery EEG subject classification in brain-machine interfaces. Biomed. Signal Process. Control.

[43] Amin S.U., Muhammad G., Abdul W., Bencherif M., Alsulaiman M. Multi-CNN feature fusion for efficient EEG classification. Proceedings of the 2020 IEEE International Conference on Multimedia & Expo Workshops (ICMEW).

[44] Roy A.M. (2022). A multi-scale fusion CNN model based on adaptive transfer learning for multi-class MI-classification in BCI system. bioRxiv.

[45] Szegedy C., Liu W., Jia Y., Sermanet P., Reed S., Anguelov D., Erhan D., Vanhoucke V., Rabinovich A. Going deeper with convolutions. Proceedings of the IEEE Conference on Computer Vision and Pattern Recognition.

[46] Chen H. (2022). Research on Spatial Cognitive Training and Evaluation Method Integrating Brain-Computer Interface and Virtual Car. Master’ Thesis.

[47] Zhong Y. (2022). Space Cognition Training System and Eeg Signal Analysis Combining Brain-Computer Interface with Virtual Drone. Master’ Thesis.

[48] Kenny B., Veitch B., Power S. (2019). Assessment of changes in neural activity during acquisition of spatial knowledge using EEG signal classification. J. Neural Eng..

[49] Yin L., Tian F., Hu R., Li Z., Yin F. (2021). Estimating Phase Amplitude Coupling between Neural Oscillations Based on Permutation and Entropy. Entropy.

[50] Dang Y., Wang Y., Xia X., Yang Y., Bai Y., Zhang J., He J. (2023). Deep brain stimulation improves electroencephalogram functional connectivity of patients with minimally conscious state. CNS Neurosci. Ther..

[51] Liang Z., Lan Z., Wang Y., Bai Y., He J., Wang J., Li X. (2023). The EEG complexity, information integration and brain network changes in minimally conscious state patients during general anesthesia. J. Neural Eng..

[52] Wen D., Jia P., Hsu S.-H., Zhou Y., Lan X., Cui D., Li G., Yin S., Wang L. (2019). Estimating coupling strength between multivariate neural series with multivariate permutation conditional mutual information. Neural Netw..

[53] Sharma N., Kolekar M.H. (2023). Dementia diagnosis with EEG using machine learning. Artificial Intelligence for Neurological Disorders.

[54] Jie H., Li S., Gang S., Albanie S. Squeeze-and-excitation networks. Proceedings of the IEEE Conference on Computer Vision and Pattern Recognition.

[55] Lun X., Yu Z., Chen T., Wang F., Hou Y. (2020). A simplified CNN classification method for MI-EEG via the electrode pairs signals. Front. Hum. Neurosci..

[56] Rakhmatulin I., Dao M.-S., Nassibi A., Mandic D. (2024). Exploring convolutional neural network architectures for EEG feature extraction. Sensors.

[57] Aldawsari H., Al-Ahmadi S., Muhammad F. (2023). Optimizing 1D-CNN-based emotion recognition process through channel and feature selection from EEG signals. Diagnostics.

[58] Zhang L., Yu Q., Zhang Y., Zhou C. (2023). Adaptive Feature Cross-Compression for Credit Default Prediction. IEEE Access.

[59] Ismail L.E., Karwowski W. (2020). Applications of EEG indices for the quantification of human cognitive performance: A systematic review and bibliometric analysis. PLoS ONE.

[60] Harmony T. (2013). The functional significance of delta oscillations in cognitive processing. Front. Integr. Neurosci..

[61] Shi E., Yu S., Kang Y., Wu J., Zhao L., Zhu D., Lv J., Liu T., Hu X., Zhang S. (2023). MEET: A Multi-Band EEG Transformer for Brain States Decoding. IEEE Trans. Biomed. Eng..

[62] Li C., Gao S., Zhang J. (2024). Multi-bands joint graph convolution EEG functional connectivity network for predicting mental disorders. Int. J. Crowd Sci..

[63] Zhu J., Shen Z., Ni T. (2022). Multi-frequent band collaborative EEG emotion classification method based on optimal projection and shared dictionary learning. Front. Aging Neurosci..

[64] Wahdow M., Alnaanah M., Fadel W., Adolf A., Kollod C., Ulbert I. (2023). Multi frequency band fusion method for EEG signal classification. Signal Image Video Process..

[65] Zhang J., Zhang X., Chen G., Zhao Q. (2022). Granger-causality-based multi-frequency band EEG graph feature extraction and fusion for emotion recognition. Brain Sci..

[66] Zarjam P., Epps J., Chen F. Characterizing working memory load using EEG delta activity. Proceedings of the 2011 19th European Signal Processing Conference.

[67] Clarke A.R., Barry R.J., McCarthy R., Selikowitz M. (1998). EEG analysis in attention-deficit/hyperactivity disorder: A comparative study of two subtypes. Psychiatry Res..

[68] Clarke A.R., Barry R.J., Heaven P.C., McCarthy R., Selikowitz M., Byrne M.K. (2008). EEG in adults with attention-deficit/hyperactivity disorder. Int. J. Psychophysiol..

[69] Babiloni C., Vecchio F., Bultrini A., Luca Romani G., Rossini P.M. (2006). Pre-and poststimulus alpha rhythms are related to conscious visual perception: A high-resolution EEG study. Cereb. Cortex.

[70] Babiloni C., Babiloni F., Carducci F., Cappa S.F., Cincotti F., Del Percio C., Miniussi C., Moretti D.V., Rossi S., Sosta K. (2004). Human cortical responses during one-bit short-term memory. A high-resolution EEG study on delayed choice reaction time tasks. Clin. Neurophysiol..

[71] Zaepffel M., Trachel R., Kilavik B.E., Brochier T. (2013). Modulations of EEG beta power during planning and execution of grasping movements. PLoS ONE.

[72] Herrmann C.S., Munk M.H., Engel A.K. (2004). Cognitive functions of gamma-band activity: Memory match and utilization. Trends Cogn. Sci..

[73] Mably A.J., Colgin L.L. (2018). Gamma oscillations in cognitive disorders. Curr. Opin. Neurobiol..

